# Age-dependent effect of high cholesterol diets on anxiety-like behavior in elevated plus maze test in rats

**DOI:** 10.1186/1744-9081-10-30

**Published:** 2014-09-01

**Authors:** Xu Hu, Tao Wang, Jia Luo, Shan Liang, Wei Li, Xiaoli Wu, Feng Jin, Li Wang

**Affiliations:** 1 Key Laboratory of Mental Health, Institute of Psychology, Chinese Academy of Sciences, Beijing, China; 2 University of Chinese Academy of Sciences, Beijing, China; 3 The affiliated Hospital, School of Medicine, Hangzhou Normal University, Zhejiang, China

**Keywords:** High cholesterol, Anxiety-like behavior, Age-dependent, Hippocampus, Corticosterone, BDNF

## Abstract

**Background:**

Cholesterol is an essential component of brain and nerve cells and is essential for maintaining the function of the nervous system. Epidemiological studies showed that patients suffering from anxiety disorders have higher serum cholesterol levels. In this study, we investigated the influence of high cholesterol diet on anxiety-like behavior in elevated plus maze in animal model and explored the relationship between cholesterol and anxiety-like behavior from the aspect of central neurochemical changes.

**Methods:**

Young (3 weeks old) and adult (20 weeks old) rats were given a high cholesterol diet for 8 weeks. The anxiety-like behavior in elevated plus maze test and changes of central neurochemical implicated in anxiety were measured.

**Results:**

In young rats, high cholesterol diet induced anxiolytic-like behavior, decreased serum corticosterone (CORT), increased hippocampal brain-derived neurotrophic factor (BDNF), increased hippocampal mineralocorticoid receptor (MR) and decreased glucocorticoid receptor (GR). In adult rats, high cholesterol diet induced anxiety-like behavior and increase of serum CORT and decrease of hippocampal BDNF comparing with their respective control group that fed the regular diet.

**Discussion:**

High cholesterol diet induced age-dependent effects on anxiety-like behavior and central neurochemical changes. High cholesterol diet might affect the central nervous system (CNS) function differently, and resulting in different behavior performance of anxiety in different age period.

## Introduction

Cholesterol is an essential component of all cell membranes and plays an important role in maintaining the structure and function of every system of the body including the nervous system [1-3]. Elevated serum cholesterol level is generally considered to be the most important risk factor for the development of cardiovascular diseases (CVDs). Recently, more and more studies have drawn growing attentions in the high co-morbidity between psychiatric disorders and CVDs [4,5]. Abnormalities in serum cholesterol levels of patients with mood disorders have also been identified in epidemiological studies. High cholesterol levels in patients with anxiety disorders have been investigated in many studies. Studies demonstrated that panic disorder patients have significantly higher serum cholesterol levels compared with age and sex-matched healthy control subjects [6]. Patients suffering from generalized anxiety disorder (GAD), obsessive compulsive disorder (OCD) and post-traumatic stress disorder (PTSD), were also found to have higher serum cholesterol levels [7-10]. However, there was poor evidence for an influence of high cholesterol diet on anxiety-like behavioral animal models. The central neurochemical relationship between cholesterol and anxiety disorders is poorly understood.

In all animals, most growth and differentiation of the CNS occurs in the first few weeks or years after birth, and cholesterol is required for these processes [11]. Studies showed that there were profound differences in the circulating cholesterol levels at different stages of development [12,13]. Meanwhile, age-dependent effect of high-fat diets on lipid metabolism and lipid accumulation was found in rats [14]. Dufour et al. examined the effect of a cholesterol-rich diet on learning performance of rats and found that the stimulating effect of cholesterol on memory retention depended on age [15]. In this study, we investigated the effects of high cholesterol diets on anxiety-like behavior tested in elevated plus maze in young (3 weeks old) and adult (20 weeks old) Sprague–Dawley rats, and explored the relationship between cholesterol and anxiety-like behavior from the aspect of central neurochemical changes.

## Materials and methods

### Animals

Male Sprague–Dawley rats, conventional clean animal grade, aged 3 weeks (young rats, n = 20) or aged 20 weeks (adult rats, n = 20), were purchased from Vital River Laboratory Animal Ltd. (Beijing, China). They were individually housed in cages and maintained at a constant temperature (23 ± 2°C) and humidity (55 ± 5%) and exposed to a 12-h light/dark cycle (lights on at 7:00 a.m.). Food and water were supplied ad libitum. All animals were cared for in accordance with the *Guide for the Care and Use of Laboratory Animals*. All experiments were approved by the Institutional Animal Care and Use Committee of the Institute of Psychology, Chinese Academy of Sciences.

### Experimental design

After 1-week adaptive period on a regular diet containing 32% (weight/weight) protein, 5% fat, 2% fiber, 1.8% calcium, 1.2% phosphorus, and a nitrogen-free extract as the remainder, young and adult rats were randomly assigned to two groups of 10 rats each. The experimental groups were as follows:

1) Young-Control (n = 10): young rats of 3 weeks old, fed regular diet for 8 weeks;

2) Young-HCD (n = 10): young rats of 3 weeks old, fed high-cholesterol diet for 8 weeks;

3) Adult-Control (n = 10): adult rats of 20 weeks old, fed regular diet for 8 weeks;

4) Adult-HCD (n = 10): adult rats of 20 weeks old, fed high-cholesterol diet for 8 weeks.

The high-cholesterol diet (HCD) contained 2% (weight/weight) cholesterol, 10% lard, 0.3% sodium cholate and 87.7% regular diet. After the feeding period, the anxiety-like behavior was tested in elevated plus maze (EPM). Twenty-four hours after the behavioral test, the rats were anesthetized with an intraperitoneally injection of sodium pentobarbital (50 mg/kg) following food deprivation for 12 hours and blood samples from each rat were collected into tubes using cardiac puncture. After blood collection, brains were rapidly removed following decapitation and the hippocampus regions were excised in ice immediately and washed in ice-cold phosphate buffer saline and then stored at -80°C until use.

### Elevated plus maze test

The elevated plus maze (EPM) is a widely used anxiety-like behavioral assay for rodents. The maze was composed of two open arms (50 × 10 cm) and two closed arms of the same size with 30-cm high walls. The whole maze was 50 cm high form the floor. The apparatus was illuminated indirectly by two lamps and light intensity was approximately 50 lx in the open arms. The tests were conducted between the 9:00–11:00 a.m. The rats were placed individually in the center zone of the maze, facing an open arm, and allowed 5 minutes of free exploration. All rats were tested just once. After each test the arena was cleaned with 70% ethanol. Time spent and distance travelled in the open and closed arms were videotaped and video recordings were analyzed by ANYmaze softmaze. An entry was scored when all four paws were in an arm of the maze. The percentage of the time spent and distance travelled in the open arms and closed arms of the maze were calculated. Locomotor activity was indexed by the total distance travelled and total number of arms entries in the entire maze during the test period.

### Assay for serum biochemical parameters

The serum was separated from the blood samples by centrifugation at 3500 rpm for 10 minutes. Total cholesterol (TC), high density lipoprotein cholesterol (HDL-C), low density lipoprotein cholesterol (LDL-C), glucose, alanine aminotransferase (ALT) and aspartate aminotransferase (AST) were measured with automatic chemical analyzer 7020 (Hitachi, Tokyo, Japan).

### Central neurochemical analysis

Serum CORT, hippocampal BDNF, serotonin receptor 1A (5-HTR1A), N-methyl-D-aspartate receptor (NMDAR), MR and GR levels and hippocampal serotonin (5-HT), dopamine (DA), noradrenaline (NA) and γ-aminobutyric acid (GABA) levels were measured using respective enzyme-linked immunosorbent assay (ELISA) kits (Cusabio Biotech Co., Ltd., Wuhan, China) according to the manufacturer’s instruction. The serum was separated from the blood samples by centrifugation at 3500 rpm for 10 minutes and then used for ELISA assay directly. The hippocampal tissues were homogenized in PBS with a glass homogenizer on ice and the resulting suspensions were subjected to two freeze-thaw cycles to further break the cell membranes. After that, the homogenates were centrifuged for 5 minutes at 5000 rpm and the supernatants were used for ELISA assay.

The microtiter plate provided in each ELISA kit has been pre-coated with an antibody specific to CORT, BDNF, 5-HTR1A, NMDAR, MR, GR, 5-HT, DA, NA or GABA. Standards or samples were added to the appropriate microtiter plate wells and incubated for 2 hours at 37°C. After removing the liquid of each well, a biotin-conjugated antibody was added to the wells and incubated. And then, horseradish peroxidase (HRP) conjugated avidin was added to the well and incubated. After TMB substrate solution was added, the wells would exhibit changes in color. The enzyme-substrate reaction was terminated by the addition of sulphuric acid solution and the color changes were measured spectrophotometrically at a wavelength of 450 nm. The concentration of the substance to be determined in the samples is then determined by comparing the O.D. value of the samples to the standard curve.

### Statistical analysis

Statistical analysis was performed using SPSS 17.0 statistical software (SPSS Inc., Chicago, IL, USA). All experimental data were presented as the mean ± S.E.M. Data were analyzed using independent t-tests. Values of p < 0.05 were considered statistically significant.

## Results

### Serum biochemical analysis

The comparison of initial and final weight, serum TC, HDL-C, LDL-C, glucose, ALT and AST in both young and adult groups after feeding high-cholesterol diet for 8 weeks was listed in Table 1. There were no significant differences in initial and final weight between control and HCD groups in both young and adult rats. It revealed that both the young and adult groups displayed the same serum biochemical changes after high-cholesterol treatment. TC, LDL-C and ALT were significantly increased (p < 0.05) and HDL-C was significantly decreased (p < 0.05) in rats fed high-cholesterol diet comparing to their respective control group that fed the regular diet. In both young and adult rats, no significant differences were observed in levels of glucose and AST between control and HCD groups.

**Table 1:** Effects of high cholesterol diets on serum biochemical parameters in young and adult rats

**Biochemical parameters**	**Young**		**Adult**	
	**Control (n = 10)**	**HCD (n = 10)**	**Control (n = 10)**	**HCD (n = 10)**
Initial weight (g)	76.57 ± 2.78	77.70 ± 2.80	553.33 ± 16.10	557.60 ± 17.48
Final weight (g)	395.81 ± 10.74	413.96 ± 7.59	610.84 ± 21.29	643.68 ± 24.40
TC (mmol/L)	1.85 ± 0.11	3.18 ± 0.18*	1.74 ± 0.08	2.38 ± 0.22*
HDL-C (mmol/L)	1.14 ± 0.06	0.95 ± 0.02*	0.97 ± 0.09	0.72 ± 0.06*
LDL-C (mmol/L)	0.42 ± 0.04	2.01 ± 0.16*	0.44 ± 0.04	1.38 ± 0.17*
Glucose (mmol/L)	8.77 ± 0.46	7.96 ± 0.58	7.78 ± 0.36	7.65 ± 0.22
ALT (U/L)	65.00 ± 4.10	94.2 ± 13.43*	62.3 ± 5.11	92.6 ± 9.55*
AST (U/L)	180.3 ± 21.04	221.5 ± 45.20	148.6 ± 15.16	160.4 ± 18.84

Control: regular diet; HC: high cholesterol diet.

The data are shown as the mean ± S.E.M. *P < 0.05 compared to the respective control group that fed the regular diet.

### Behavioral test

**Figure 1:**
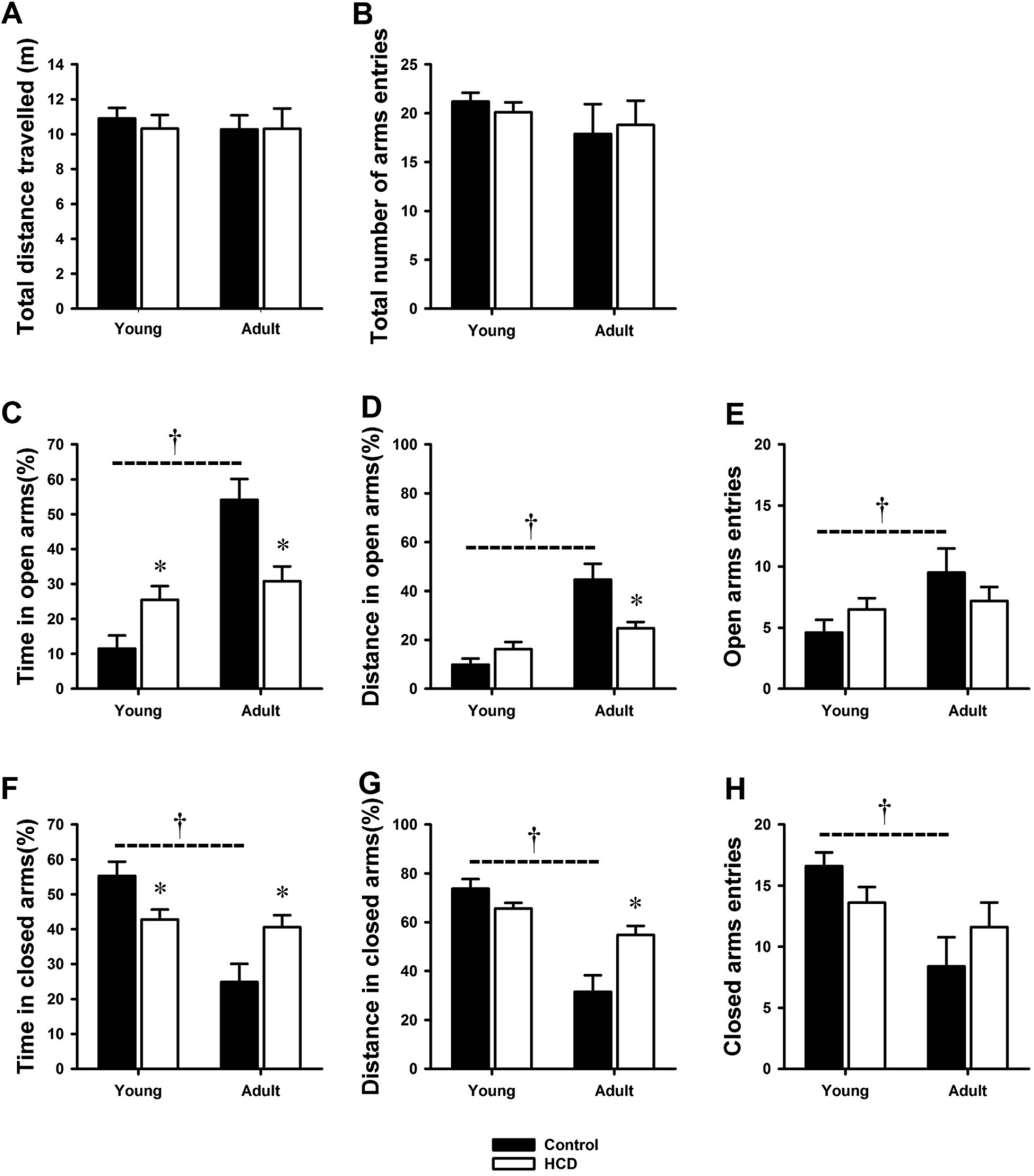
**Effects of high cholesterol diet on anxiety-like behavior in elevated plus maze test in young and adult rats. (A)** Total distance travelled in elevated plus maze; **(B)** Total number of arms entries in entire maze; **(C)** Time spent in open arms; **(D)** Distance travelled in open arms; **(E)** Number of open arms entries; **(F)** Time spent in closed arms; **(G)** Distance travelled in closed arms; **(H)** Number of closed arms entries. The data are shown as the mean ± S.E.M. [n = 10 for each group; Control: regular diet; HCD: high cholesterol diet; *P < 0.05 compared to the respective control group that fed the regular diet. †P < 0.05 compared between Young-Control and Adult-Control group].

There were no significant differences between control and HCD rats in total distance travelled and total number of arms entries in EPM in both young and adult groups (Figure 1A and 1B). These suggested that there were no differences in locomotor activity among groups. In young rats, it showed a significant increase in time spent in open arms and a significant decrease in time spent in closed arms in HCD group compared to control group (Figure 1C and 1F). However, there were no significant difference between HCD and control group in distance travelled in open and closed arms (Figure 1D and 1G). There were also no significant differences in number of open and closed arms entries between HCD and control group in young rats (Figure 1E and 1H). Different results were found in adult rats. In adult rats, a significant decrease in time spent in open arms and a significant increase in time spent in closed arms were found in HCD group compared to control group (Figure 1C and 1F). Simultaneously, the distance travelled in open arms was also significantly decreased and distance in closed arms was significantly increased in HCD group (Figure 1D and 1G). No significant differences were found between HCD and control group in number of open and closed arms entries in adult rats (Figure 1E and 1H). These results suggested that the HCD group displayed anxiolytic-like behavior in young rats and displayed anxiety-like behavior in adult rats comparing to their respective control group that fed the regular diet. We also compared the anxiety-like behavior in control group between young and adult rats. The Adult-Control rats showed less anxiety-like behavior than Young-Control rats. There were significant increases in time spent, distance travelled and number of entries in open arms and significant decreases in time spent, distance travelled and number of entries in closed arms in Adult-Control rats (Figure 1C, 1D, 1E, 1F, 1G and 1H).

### Serum CORT

**Figure 2:**
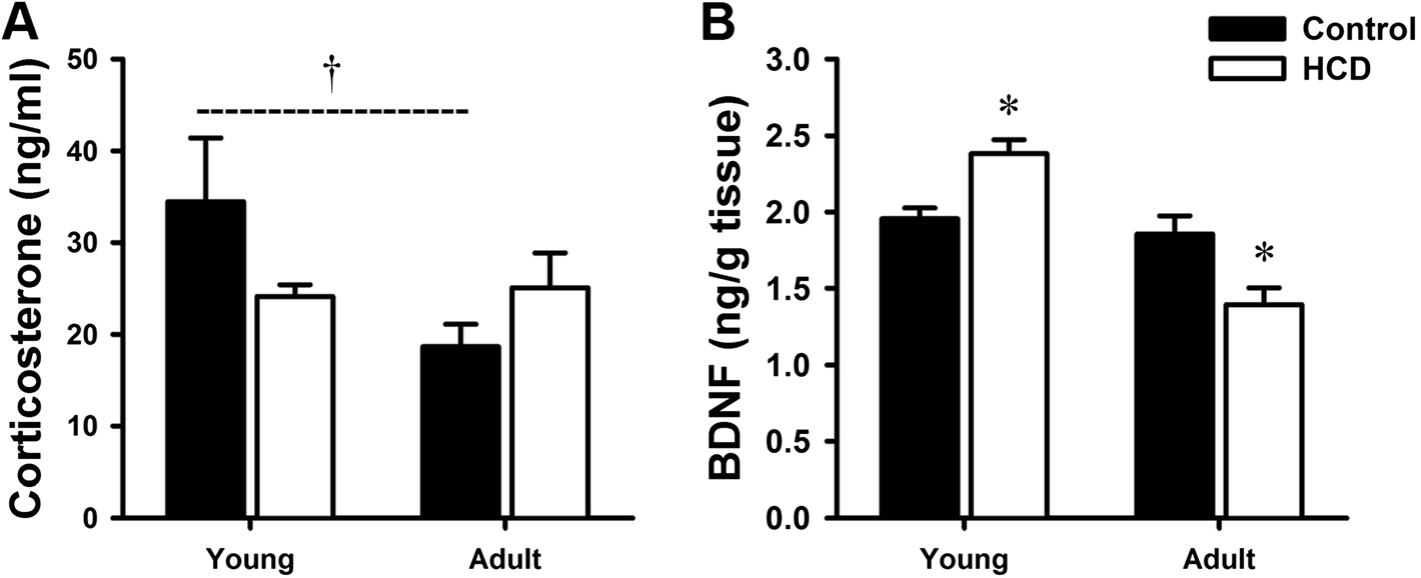
**Effects of high cholesterol diet on serum corticosterone and hippocampal BDNF levels in young and adult rats. (A)** Serum corticosterone levels; **(B)** Hippocampal BDNF levels. The data are shown as the mean ± S.E.M. [n = 10 for each group; Control: regular diet; HCD: high cholesterol diet; BDNF: brain-derived neurotrophic factor; *P < 0.05 compared to the respective control group that fed the regular diet. †P < 0.05 compared between Young-Control and Adult-Control group].

The CORT levels were decreased in young rats and increased in adult rats after HCD treatment compared to their respective control group (Figure 2A). However, there were no statistical significances both in young and adult rats (Young rats: 34.46 ± 6.96 ng/ml *vs.* 24.16 ± 1.22 ng/ml, p = 0.118; Adult rats: 18.67 ± 2.45 ng/ml vs. 25.06 ± 3.79 ng/ml, p = 0.194). But the serum CORT levels in Adult-Control rats were significantly less than Young-Control rats (34.46 ± 6.96 ng/ml vs. 18.67 ± 2.45 ng/ml, p = 0.044).

### Altered hippocampal BDNF levels

In young rats, HCD group showed increases in hippocampal BDNF levels compared to control group (p = 0.002). Reversely, in adult rats, HCD group showed decreases in hippocampal BDNF levels (p = 0.013) (Figure 2B). But, there were no significant changes between Young-Control rats and Adult-Control rats (p = 0.479).

### Neurotransmitters in hippocampus

**Figure 3:**
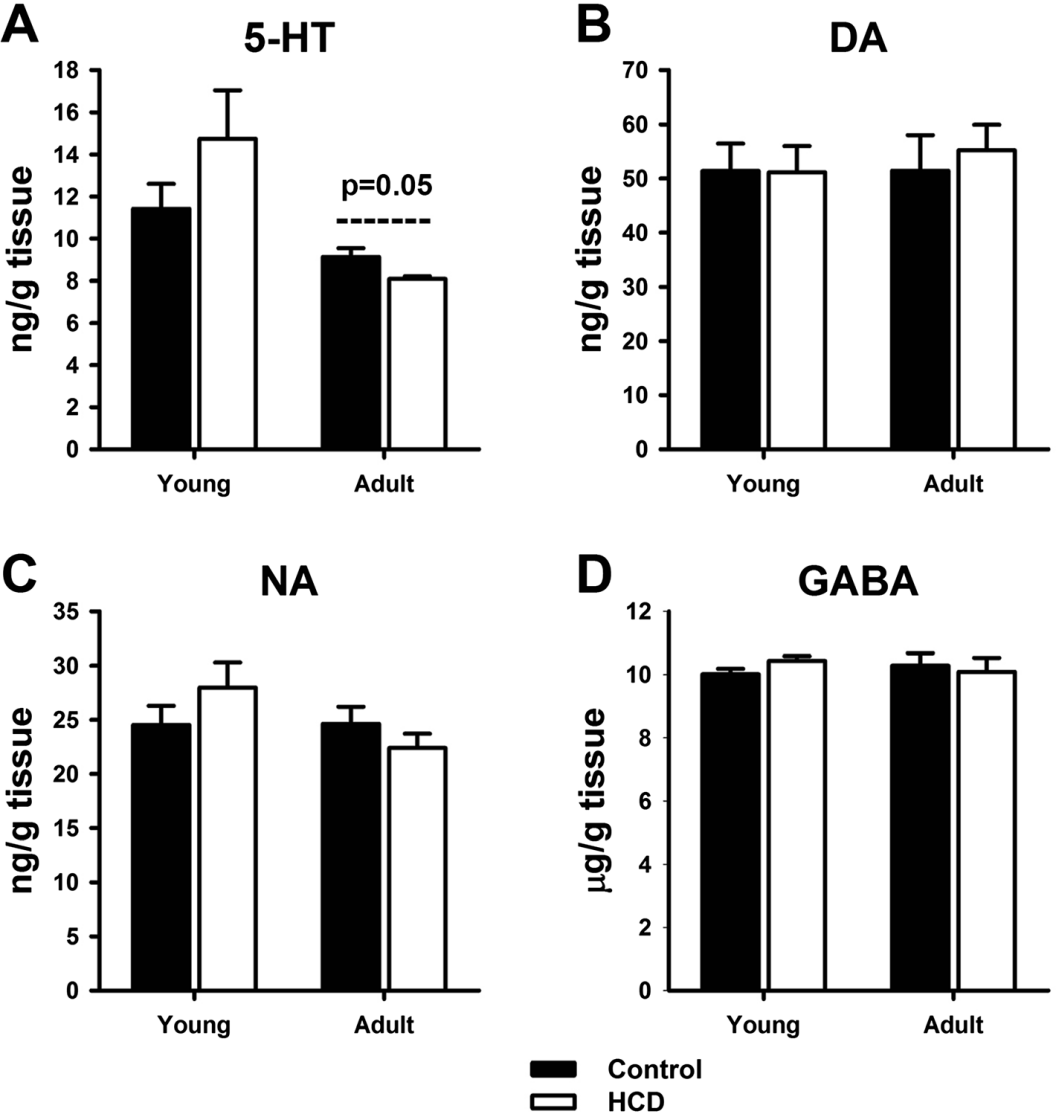
**Effects of high cholesterol diet on neurotransmitters in hippocampus in young and adult rats. (A)** Hippocampal 5-HT levels; **(B)** Hippocampal DA levels; **(C)** Hippocampal NA levels; **(D)** Hippocampal GABA levels. The data are shown as the mean ± S.E.M. [n = 10 for each group; Control: regular diet; HCD: high cholesterol diet; 5-HT: serotonin; DA: dopamine; NA: noradrenaline; GABA: γ-aminobutyric acid].

There were no significant alterations in 5-HT, DA, NA and GABA levels in young rats after HCD treatment (Figure 3). In adult rats, there were also no significant differences in DA, NA and GABA levels between control and HCD groups. The 5-HT levels were obviously reduced in Adult-HCD rats compared to Adult-Control rats (Figure 3A, p = 0.05).

### Changes of 5-HTR1A, NMDAR, MR, GR in hippocampus

**Figure 4:**
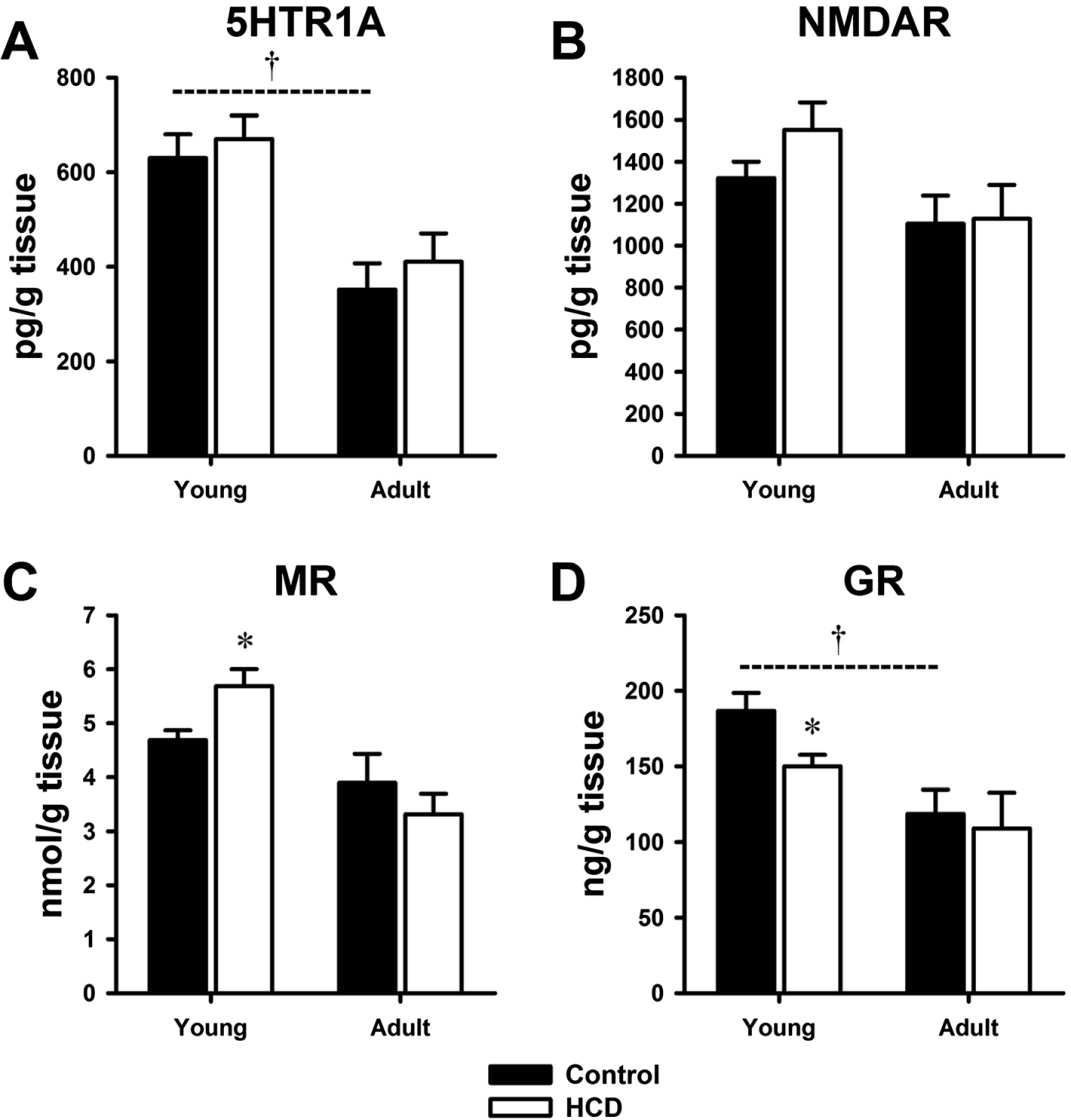
**Effects of high cholesterol diet on hippocampal 5-HTR1A, NMDAR, MR, GR levels in young and adult rats. (A)** Hippocampal 5-HTR1A levels; **(B)** Hippocampal NMDAR levels; **(C)** Hippocampal MR levels; **(D)** Hippocampal GR levels. The data are shown as the mean ± S.E.M. [n = 10 for each group; Control: regular diet; HCD: high cholesterol diet, 5-HTR1A: serotonin receptor; NMDAR: N-methyl-D-aspartate receptor; MR: mineralocorticoid receptor; GR: glucocorticoid receptor. *P < 0.05 compared to the respective control group that fed the regular diet. †P < 0.05 compared between Young-Control and Adult-Control group].

In young rats, MR levels were significantly increased (Figure 4C, p = 0.020) and GR levels were significantly decreased (Figure 4D, p = 0.026) in HCD group compared to control group. The 5-HTR1A and NMDAR levels were not altered in young rats after HCD treatment (Figure 4A and 4B). In adult rats, we did not observed any significant changes in hippocampal 5-HTR1A, NMDAR, MR and GR levels in HCD group comparing to control group. We also compared the changes of 5-HTR1A, NMDAR, MR, GR levels between Young-Control and Adult-Control rats. The hippocampal 5-HTR1A and GR levels in Adult-Control rats were significantly lower than Young-Control rats (5-HTR1A: p = 0.002; GR: p = 0.004) (Figure 4A and 4D).

## Discussion

The incidence of hypercholesterolemia is increasing rapidly with the improvements of people’s living standards and alterations of lifestyles. Hypercholesterolemia is strongly associated with cardiovascular diseases (CVDs), such as hypertension, coronary heart disease, diabetes [16,17], and there were lots of evidences linking anxiety to CVDs [5]. However, few studies have examined the relationship of serum cholesterol levels and anxiety-like behaviors in animal models. In this study, we investigated the effects of high cholesterol diets on anxiety-like behavior tested in elevated plus maze in young and adult rats, and explored the possible relationship between cholesterol and anxiety-like behavior from the aspect of central neurochemical changes.

High cholesterol diets gave rise to increased TC, LDL-C, ALT and decreased HDL-C levels in both young and adult rats. The similar results were also reported in our previous study [18]. However, the effects of high cholesterol diet on anxiety-like behavior displayed reverse result in young and adult rats. The young rats displayed anxiolytic-like behavior and adult rats displayed anxiety-like behavior after high cholesterol diet treatment comparing to their respective control fed regular diet. These results suggested that the effects of high cholesterol diet on anxiety-like behavior might depend on the age of the animal. Cholesterol might play an important role in the different performance. The brain has a relatively self-sufficient supply and transport of cholesterol [1]. However, there were evidences suggested that circulating cholesterol might be transported to the brain via the transcytosis of low-density lipoprotein (LDL) across the blood–brain barrier by LDL receptor [19]. Thus, the brain cholesterol level might be determined by the endogenous cellular de novo synthesis and external supply. Therefore, high cholesterol diet might result in an increase of brain cholesterol content. In the first few weeks or years after birth, cholesterol supplement in this development period might be beneficial for the growth and differentiation of the CNS. Therefore, high cholesterol diet reduced the anxiety-like behavior in young rats. In adult rats, the growth and differentiation of CNS is mature, and high cholesterol diet induced excessive accumulation of cholesterol might affect brain function. So, the high cholesterol diet induced anxiety-like behavior in adult rats compared to Adult-Control rats. Meanwhile, we found that the rats displayed less anxiety-like behaviors in controls with the age increasing.

Studies have provided evidence for an association between Hypothalamus-Pituitary- Adrenal (HPA) axis function and psychiatric problems [20]. The HPA axis becomes active in response to stress and increased levels of anxiety is associated with activated HPA axis functions. HPA axis activation is assessed by measuring levels of serum CORT [21]. In this study, we found that serum CORT levels decreased 29.89% in young rats and increased 34.23% in adult rats after high cholesterol diets treatment compared to their respective control groups, although there were no statistical significances. This suggested that HPA axis might be participated in the high cholesterol diets induced changes of anxiety-like behavior in young and adult rats. Meanwhile, we found that Adult-Control rats displayed less anxiety than Young-Control rats, which was also accompanied by the reduction of serum CORT levels in Adult-Control rats comparing with Young-Control rats.

Evidence from preclinical and clinical literature suggested that brain-derived neurotrophic factor (BDNF) in the hippocampus influenced stress-related behaviors and these studies had demonstrated that impaired BDNF signaling in the dentate gyrus of mice resulted in a marked increase in anxiety-like behavior [22,23]. Restraint stress was known to decrease expression of hippocampal BDNF, activate the HPA axis, and result in increased anxiety-like behavior in rodents [24-26]. These reports suggested that lower BDNF levels were related to increased anxiety-like behavior. In young rats, high cholesterol diet induced less anxiety-like behavior and significantly increased hippocampal BDNF level. Adult rats displayed anxiety-like behavior and accompanied by decreased hippocampal BDNF level after high cholesterol diet treatment. So, our study supported the previous reports further and BDNF signaling might play important roles in high cholesterol diets induced changes of anxiety-like behavior in rats. However, the different performance between young and adult control rats seems unrelated to BDNF signaling because there were no significant changes between young and adult control rats.

Serotonin, dopamine, noradrenaline and γ-aminobutyric acid are neurotransmitters which have been proposed to be involved in the pathogenesis of anxiety, depression and mood control [27-30]. We analyzed the levels of these neurotransmitters in hippocampus. There were no significant alterations in serotonin, dopamine, noradrenaline and γ-aminobutyric acid levels in both young and adult rats after high-cholesterol diet treatment. Only the serum serotonin level in adult rats were slightly reduced in high cholesterol treatment group (p = 0.05). These results suggested that the high cholesterol diets induced changes of anxiety-like behavior might be not accompanied by changes in specific neurotransmitters.

Serotonin receptors are distributed throughout the CNS and several receptors have been implicated in anxiety-like behavior, especially the 5HT1A receptor has received more attention [31,32]. NMDA receptors are known to play an important role in synaptic development and plasticity and are thought to be involved in anxiety-like behavior [33]. NMDA receptor antagonists are known to block anxiety in both mice and rats [34]. In this study, we did not observed any significant changes in hippocampal 5-HT1A and NMDA receptor after high cholesterol diet treatment both in young and adult rats.

CORT regulates stress response and influence emotion in the brain which are mediated by the high-affinity mineralocorticoid receptor (MR) and low-affinity glucocorticoid receptor (GR) [35]. MR has a tenfold higher affinity than GR, so hippocampal MR responds strongly to CORT. The predominant activation of the MR at a low CORT level and additional activation of the GR during increased CORT level could cause the alterations of neuronal integrity and functions responding to stress, such as changes in neuroendocrine regulation and behavior [36]. MR is an important regulator of HPA axis functions and deletion of MR results in increased serum CORT levels [37]. Continuous predominant MR activation appears to be beneficial for the emotional state, resulting in low anxiety [38]. In young rats, MR levels were significantly increased in high cholesterol diet group compared to Young-Control group, so the young rats displayed less anxiety-like behavior after high cholesterol treatment. GR becomes active as CORT release increases, mediating behavioral activation and providing negative feedback inhibition to the HPA axis [39]. In our study, serum CORT level had a tendency to decline (29.89% decreased) after high cholesterol diet treatment in young rats and GR level was also significantly decreased. Meanwhile, the serum CORT was significantly decreased in adult rats compared to young rats and accompanied by GR level decreased. In adult rats, there were no significant alterations of MR and GR between control and high cholesterol diet group. These results suggested that coordinative reaction of MR and GR may be participated in the high cholesterol induced anxiolytic-like behavior in young rats.

## Conclusions

After feeding high cholesterol diet for 8 weeks, the rats displayed age-dependent effects on anxiety-like behavior in elevated plus maze test and central neurochemical changes. High cholesterol diet induced anxiolytic-like behavior in young rats and high cholesterol diet induced anxiety-like behavior in adult rats comparing to their respective control group. The different performance of anxiety-like behavior was accompanied by different central neurochemical changes in young and adult rats. In young rats, decreased serum CORT, increased hippocampal BDNF, increased hippocampal MR and decreased GR were observed in high cholesterol group compared to Young-Control. In adult rats, high cholesterol diet induced the increase of serum CORT and decrease of hippocampal BDNF comparing with Adult-Control. High cholesterol diet would affect the CNS function differently, resulting in different behavior performance of anxiety in different age period.

## Abbreviations

CORT: Corticosterone; BDNF: Brain-derived neurotrophic factor; MR: Mineralocorticoid receptor; GR: Glucocorticoid receptor; CNS: Central nervous system; CVDs: Cardiovascular diseases; GAD: Generalized anxiety disorder; OCD: Obsessive compulsive disorder; PTSD: Post-traumatic stress disorder; HCD: High cholesterol diet; EPM: Elevated plus maze; TC: Total cholesterol; HDL-C: High density lipoprotein cholesterol; LDL-C: Low density lipoprotein cholesterol; ALT: Alanine aminotransferase; AST: Aspartate aminotransferase; 5-HTR1A: Serotonin receptor; NMDAR: N-methyl-D-aspartate receptor; 5-HT: Serotonin; DA: Dopamine; NA: Noradrenaline; GABA: γ-aminobutyric acid.

## Competing interests

The authors declare that they have no competing interests.

## Authors' contributions

All authors listed have contributed to the work. HX and WT participated in designing the experimental protocol, data collection, statistical analysis and writing the manuscript. JF and WL designed and supervised the study and revised the manuscript. LJ, LS, LW and WXL carried out for behavioral test and sample collection. HX and WT contributed to the work equally and should be regarded as co-first authors. JF and WL should be regarded as co-corresponding authors. All authors read and approved the final manuscript.
